# Modulation of Calretinin Expression in Human Mesothelioma Cells Reveals the Implication of the FAK and Wnt Signaling Pathways in Conferring Chemoresistance towards Cisplatin

**DOI:** 10.3390/ijms20215391

**Published:** 2019-10-29

**Authors:** Janine Wörthmüller, Valérie Salicio, Anne Oberson, Walter Blum, Beat Schwaller

**Affiliations:** 1 Anatomy, Section of Medicine, University of Fribourg, Route Albert-Gockel 1, 1700 Fribourg, Switzerland; janine.woerthmueller@unifr.ch (J.W.); valerie.salicio@unifr.ch (V.S.); anne.oberson@unifr.ch (A.O.); 2 Genetica AG, 8001 Zurich, Switzerland; walti.blum@gmail.com

**Keywords:** malignant mesothelioma, IPTG-inducible system, calretinin, FAK signaling, Wnt signaling, chemoresistance, cisplatin

## Abstract

Malignant mesothelioma (MM) is an aggressive asbestos-linked neoplasm, characterized by dysregulation of signaling pathways. Due to intrinsic or acquired chemoresistance, MM treatment options remain limited. Calretinin is a Ca^2+^-binding protein expressed during MM tumorigenesis that activates the FAK signaling pathway, promoting invasion and epithelial-to-mesenchymal transition. Constitutive calretinin downregulation decreases MM cells’ growth and survival, and impairs tumor formation in vivo. In order to evaluate early molecular events occurring during calretinin downregulation, we generated a tightly controlled IPTG-inducible expression system to modulate calretinin levels in vitro. Calretinin downregulation significantly reduced viability and proliferation of MM cells, attenuated FAK signaling and reduced the invasive phenotype of surviving cells. Importantly, surviving cells showed a higher resistance to cisplatin due to increased Wnt signaling. This resistance was abrogated by the Wnt signaling pathway inhibitor 3289-8625. In various MM cell lines and regardless of calretinin expression levels, blocking of FAK signaling activated the Wnt signaling pathway and *vice versa*. Thus, blocking both pathways had the strongest impact on MM cell proliferation and survival. Chemoresistance mechanisms in MM cells have resulted in a failure of single-agent therapies. Targeting of multiple components of key signaling pathways, including Wnt signaling, might be the future method-of-choice to treat MM.

## 1. Introduction

Malignant mesothelioma (MM) is an aggressive cancer that arises from mesothelial cells covering the surfaces of the pleura, peritoneum, and pericardium, and is typically associated with exposure to asbestos fibers [1]. Due to the late stage at which most patients are diagnosed and the multiple genetic alterations occurring during the latency period [2], together with the high invasiveness and intrinsic chemoresistance acquired by the transformed MM cells, systemic treatment generally results in merely short-term regression and rapid local tumor relapse [3]. Currently, a multimodal treatment regimen of chemotherapy consisting in cisplatin (Cis-Pt) plus pemetrexed, surgery, and radiotherapy provide the best long-term results [4]. However, even after such an aggressive approach, the median overall survival time is less than 12 months [5]. In recent times, also with the possibility of massively parallel sequencing strategies, a more genome-wide view on the genetic landscape of MM is slowly emerging [6]. This is expected to lead to the identification of several altered genes and signaling pathways that might serve to identify potential novel candidates for specific MM-targeted approaches [7]. 

Cancer cells develop resistance to chemotherapy primarily by inactivating apoptotic factors and/or enhancing cell survival pathways [8]. However, the mechanisms leading to chemoresistance are not yet fully understood; it might be associated with several mechanisms and discussed anomalously activated pathways include the PI3K/AKT, focal adhesion kinase (FAK) and Wnt/β-catenin signaling pathways [9,10,11]. In addition to its roles in the regulation of various cellular processes, including cell proliferation, survival, migration, and polarity [12], the dysregulated Wnt/β-catenin signaling pathway contributes to tumorigenesis and recurrence, as well as to an enhanced potency of cancer stem cells (CSCs) and resistance to anticancer therapies in many types of cancers, including MM [13,14]. Moreover, aberrant Wnt/β-catenin signaling has been implicated in decreased patient survival [15]. Wnt5a, an essential component of this pathway, increases the resistance of lung cancer cells to Cis-Pt through the activation of the Wnt5a/PKC signaling pathway [16]. In addition, c-Jun N-terminal kinase (JNK), a protein involved in determining the cell fate and promoting resistance to Cis-Pt-induced apoptosis in several human tumors [17], is also activated by Wnt5a [18]. Consequently, there are major efforts to develop new therapeutic approaches to target this pathway [19].

The Ca^2+^-binding protein calretinin (CR) has been suggested to play a role in the initiation process of mesotheliomagenesis, since CR expression is elevated in reactive mesothelial cells [20], assumed to be the starting point for the subsequent transformation into mesothelioma cells. CR was the first identified positive marker to distinguish CR-positive (CR^+^) epithelioid and biphasic MM from adenocarcinoma [21,22], and nowadays is still part of a panel of antibodies for MM identification [23]. Moreover, in transformed CR^+^ MM cells CR was shown to promote migration and invasion and to be implicated in the process of epithelial-to-mesenchymal transition (EMT) in human MM cell lines in vitro [24]. These effects are mediated through increased activation of the FAK signaling pathway, a pathway well known to orchestrate malignancy by regulating tumorigenesis and metastasis [25]. In addition, CR overexpression protects immortalized mesothelial cells from acute asbestos-induced cytotoxicity in vitro leading to an increased survival of asbestos-exposed CR-expressing mesothelial cells, proposed to promote and favor MM development [26].

Conversely, constitutive and rapid CR downregulation via a lentiviral sh*RNA* against CR (*CALB2*) results in blocking of proliferation and decreased survival of CR^+^ MM cells including MSTO-211H and ZL55 MM cells in vitro [27]. Moreover MSTO-211H cells with inhibited CR expression show a significantly reduced tumor formation capacity in vivo as demonstrated in an orthotopic mouse model [24]. Hence, CR might be considered as a potential and promising therapeutic target for MM treatment of CR^+^ MM, i.e., of MM mostly of the epithelioid and bi-phasic histotype.

The development of an in vitro system, where CR expression levels may be regulated in a precisely controlled manner might be viewed as a strategy to investigate early effects on different signaling pathways caused by CR downregulation. It might also serve to unraveling the complex crosstalk likely existing between different signaling pathways. Moreover, this approach is considered as a promising tool to manipulate (downregulate) CR protein levels in vivo at early stages of MM development, thus allowing to monitoring cancer progression within the tumor microenvironment.

## 2. Results

### 2.1. Generation of a shRNA-IPTG Inducible System to Downregulate CR

**Figure 1:**
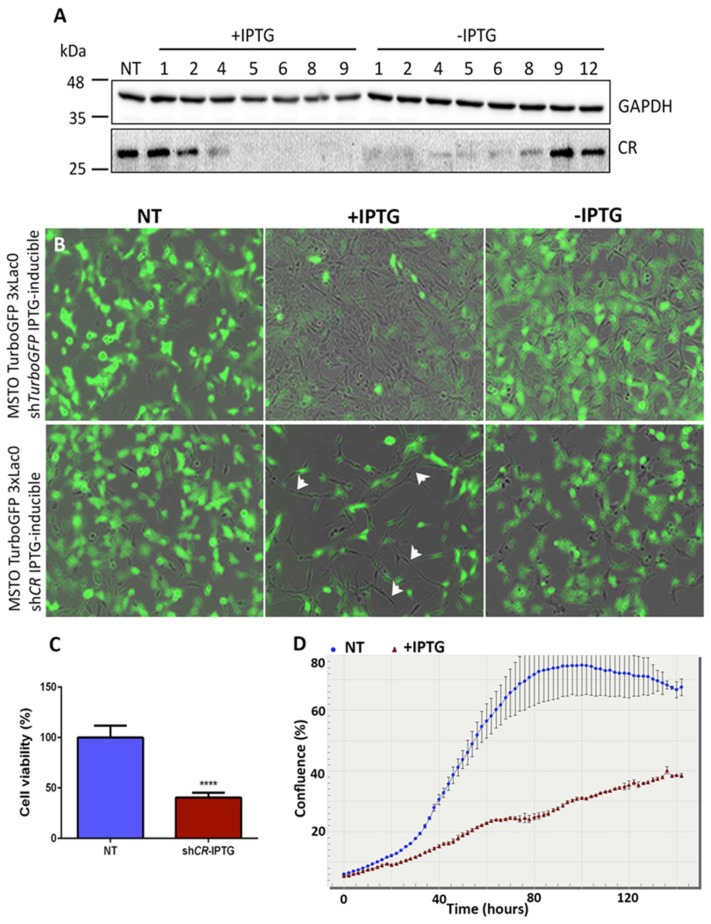
CR downregulation with a *shCALB2-*IPTG inducible system. (**A**) Western blot analyses of extracts from MSTO-shCALB2-IPTG cells induced with IPTG for nine days (+IPTG) and after IPTG removal (-IPTG) up to 12 days. GAPDH was used as loading control. (**B**) Images of MSTO-shCALB2-IPTG induced cells compared with MSTO-shTurboGFP (wt; control) cells; images are overlays of brightfield and fluorescence images. Untreated cells are composed of both, sarcomatoid (spindle-shaped) and epithelioid cells. Addition of IPTG to MSTO-wt cells (upper part) reduces green fluorescence in many cells without affecting cell proliferation and cell morphology. IPTG removal (-IPTG) restores the green fluorescence. Addition of IPTG to MSTO-shCALB2-IPTG cells considerably decreases cell numbers and the few surviving cells are mainly composed of spindle-like cells (lower panel, arrowheads). IPTG removal reverts morphology to the pre-IPTG state with the characteristic biphasic morphology (majority of cells display epithelioid morphology). (**C**) Cell number/viability, determined by an MTT assay shows a decrease in the number of viable MSTO-shCALB2-IPTG cells by approximately 60% (*p* ≤ 0.0001) after nine days of IPTG treatment when compared with non-treated cells. (**D**) Real-time growth curves of MSTO-shCALB2-IPTG cells treated with IPTG (**red**) or grown in absence of IPTG (**blue**).

With the aim to control CR expression in a temporally precise and inducible way in MM cells, we generated a pLKO-puro-IPTG-3xLacO inducible *Calb2* sh*RNA* using commercially available plasmids (see Methods). MSTO-211H cells, a commonly used MM cell line for in vitro and in vivo studies, were initially transduced with a lentivirus leading to the constitutive expression of TurboGFP. These green MSTO-211H cells allowed to investigating in real-time the knockdown of TurboGFP using the IPTG-inducible sh*RNA* system targeting *TurboGFP* mRNA. In the same cells the IPTG-inducible sh*CALB2* lentiviral system was tested; the sh*CALB2* sequence was derived from a previously validated sh*RNA* sequence shown to effectively downregulate CR in MM cells with no discernable off-target effects [27]. To assess the efficacy, TurboGFP-expressing MSTO-211H cells additionally containing either the pLKO-puro-IPTG-3xLacO inducible *Calb2* or *TurboGFP* sh*RNA* system were cultured in the presence of IPTG (1 mM) for 9 days and tracked with the time-lapse microscope IncuCyte™ system that monitors cell proliferation (confluence) and measures fluorescence intensity. In addition, CR protein levels were analyzed at different time points by Western blot analyses in the continuous presence of IPTG, but also after IPTG removal. CR protein levels decreased visibly at day 2 of IPTG treatment and from day 5 on CR levels were consistently decreased by around 90−95% (Figure 1A). CR levels started to increase four days after IPTG removal and were completely restored to pre-IPTG (basal) levels at day 9 of IPTG withdrawal. These experiments demonstrate the rather rapid IPTG-induced decrease in CR expression to residual levels generally lower than 10% of the initial levels and somewhat slower recovery to pre-IPTG levels after IPTG removal, thus indicating that the pLKO-puro-IPTG-3xLacO inducible *Calb2* sh*RNA* system is fully reversible in vitro.

The sh*CALB2*-IPTG-inducible system was additionally tested in the (epithelioid) MM cell line ZL55, expressing similar CR levels as MSTO-211H cells (Figure S1A). IPTG treatment considerably decreased CR levels as shown after 8 and 11 days of treatment; subsequent IPTG removal resulted in a strong CR re-expression observable already 3 days after IPTG removal (Figure S1B).

### 2.2. Inducible CR Downregulation Leads to a Decrease in the Proliferation and Survival of MSTO-211H Cells

In TurboGFP-expressing MSTO-211H cells, IPTG treatment of cells transduced with the sh*TurboGFP* lentivirus showed a decrease in the green fluorescence at day 9 of IPTG induction without changes of the cell morphology (Figure 1B) and also without apparent changes in cell growth. The cell morphology of the few remaining green cells and of the majority of essentially non-green cells was indistinguishable. After IPTG removal green fluorescence reappeared and at day 9 of IPTG removal was similar to control levels of cells without IPTG treatment. Of importance, no differences at the protein level of CR or of the proliferation rate were detected after IPTG induction and after IPTG removal. Regarding the MSTO-shCALB2-IPTG (induced) cells, a clear reduction in the cell number (when compared to the control shTurboGFP-IPTG cells) without changes of the green fluorescence was evident at day 9 of IPTG induction (Figure 1B). Moreover, in cells with downregulated CR expression, the cell morphology was altered; the remaining cells were more spindle-like (sarcomatoid) in the presence of IPTG. The typical morphology of MSTO-211H cells was recovered after IPTG removal, i.e., a majority of cells showed epithelioid morphology and only few cells had a more spindle-like morphology. Cell proliferation and viability assays (MTT) confirmed a decrease in the proliferation rate and number of viable cells by approximately 60% (*p < 0.0001) determined at day 9 of IPTG treatment when compared to non-treated cells (Figure 1C,D). A similar, yet smaller effect (30% MTT signal decrease) was also detected in ZL55-shCALB2-IPTG (induced) cells (Figure S1C). This corresponds well with previous results on constitutive CR downregulation, where a stronger reduction in cell growth/viability was observed in MSTO-211H compared to ZL55 cells [27]. The larger effect in MSTO-211H prompted us to preferentially use this cell line for further experiments aimed at elucidating CR’s impact in MM cells. The increased apoptosis in MSTO-211H and ZL55 cells after constitutive CR depletion by a sh*CALB2* approach had already been shown before and was found to be mediated via the indirect (intrinsic) caspase 9-dependent pathway [27]. However, due to the rapid effect of constitutive lentiviral-mediated sh*CALB2* CR downregulation (most cells entering apoptosis 72 h post-infection) the analysis of different signaling pathways modulated by CR downregulation could not be investigated. Thus, the novel IPTG-inducible approach characterized by slower kinetics of CR downregulation allowed to analyzing the different molecular events occurring during CR downregulation and recovery after IPTG removal. Since MSTO-shTurboGFP-IPTG (induced) cells showed no changes at the level of cell morphology, proliferation rate, viability, or differential protein expression levels, for the next series of experiments either MSTO-shTurboGFP-IPTG (induced; short name MSTO-wt), or non-induced MSTO-shCALB2-IPTG cells were indistinctly used as controls.

### 2.3. CR Downregulation Impairs Migration and Invasion in Vitro

**Figure 2:**
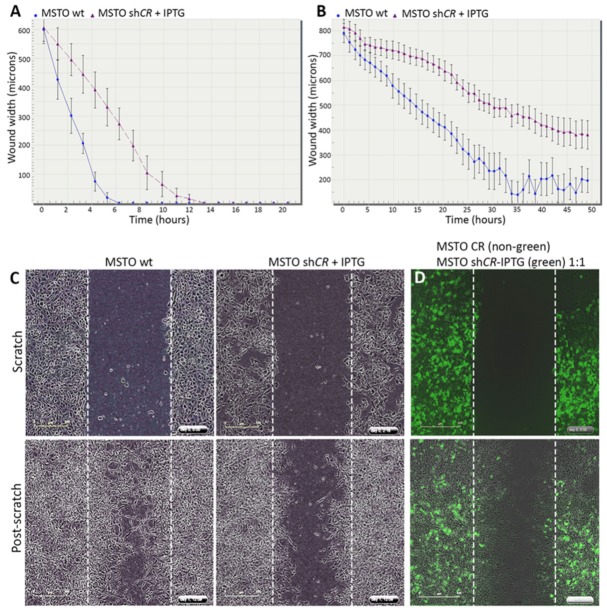
CR downregulation decreases migration and invasion of MSTO-211H cells. (**A**) Comparison of scratch closure kinetics (migration) between MSTO-wt (**blue**) and MSTO-shCALB2-IPTG (induced) cells (**purple**) monitored with the IncuCyte™ imaging system every 2 h. (**B**) Invasiveness was determined by a modified ‘scratch wound assay’ with cells required to cross a matrix barrier component (Matrigel™); comparison of wound closure kinetics between MSTO-wt (**blue**) and MSTO-shCALB2-IPTG cells measured up to 50 h. (**C**) Brightfield images were taken after confluent monolayers of MSTO-wt and MSTO-shCALB2-IPTG (induced) cells were scratched (time point *t* = 0 h, upper), and after 36 h (post-scratch, lower). Sh*CALB2*-IPTG (induced) cells show decreased invasive capacity when compared to MSTO-wt cells. (**D**) CR-overexpressing MSTO cells (non-fluorescent), shown to have an increased invasive phenotype [24], and MSTO-shCALB2-IPTG (induced) cells (**green**) were mixed at a ratio of 1:1. The pre-scratch situation is shown in the upper panel. After complete wound closure (post-scratch, lower panel), the gap is mostly filled (invaded) with non-fluorescent MSTO-CR cells. Scale bar: 400 µm.

As CR is known to increase migration and invasion of MSTO-211H cells in vitro [24], MSTO-shCALB2-IPTG (induced) cells were used to investigate, whether a near lack of CR would affect the cell’s migratory and invasive capacity. Migration was tested in the prototypical scratch wound assay. A scratch of about 1 mm was made and the closure of the wound was measured using the time-lapse Incucyte™ imaging system. After IPTG induction, cells with decreased CR levels showed a slower migration rate (closure of the scratch after 12-13 h), when compared with MSTO-wt cells (showing an average of 6 h for total wound closure; Figure 2A). Cell invasion was measured as the ability of the cells to invade a Matrigel-filled gap (0.6 mg/mL Matrigel) generated in the scratch wound assay. Neither MSTO-wt nor MSTO-shCALB2-IPTG (induced) cells were able to completely close the wound after 50 h (Figure 2B); nevertheless, the latter cells were discernibly characterized by a decreased invasive phenotype/capacity compared to the corresponding MSTO-wt cells that almost completely closed the gap by invasion through the Matrigel matrix (Figure 2C).

To further validate CR as a direct inducer of augmented invasion, a scratch was made in a confluent layer initially consisting of non-fluorescent MSTO-CR overexpressing cells and TurboGFP-labeled shCALB2-IPTG (induced) cells at a ratio of 1:1. After complete gap closure, the initially cell-devoid region was mostly invaded by non-fluorescent MSTO-CR (CR-overexpressing) cells. This further supports the notion that the presence of CR promotes invasion, as shown previously [24], while the downregulation of CR (induced by IPTG) decreases the invasive capacity of MSTO-211H cells (Figure 2D, Movie Supplementary Movie S1).

### 2.4. MM Cells with Decreased Levels of CR Show A Decrease in FAK and p-FAK (Tyr_397_) Levels and An Increased Sensitivity Towards the FAK Inhibitor VS-6063

**Figure 3:**
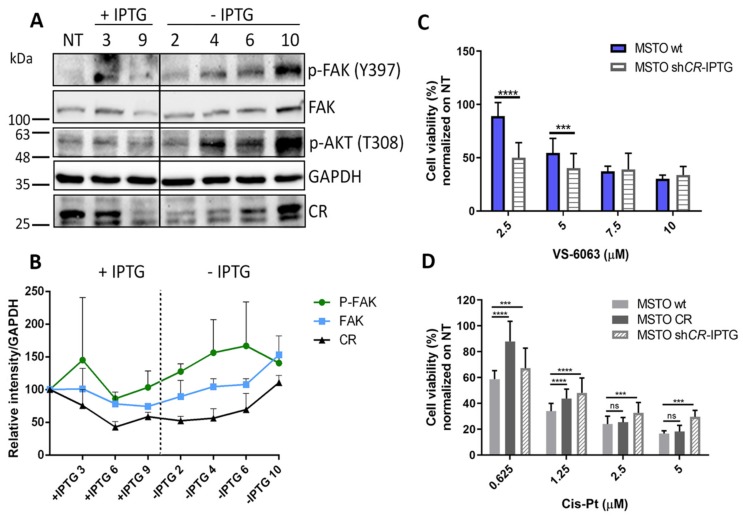
Effect of CR downregulation on different proteins and cell viability after treatment with VS-6063 and Cis-Pt. (**A**) Protein levels of p-FAK (Tyr_397_), FAK, p-Akt (Thr_308_) and CR measured at different time points during IPTG induction and IPTG removal. Levels of p-FAK (Tyr_397_) and FAK are decreased after 9 days of IPTG induction and subsequent CR depletion; levels are restored after IPTG removal and CR increases to pre-IPTG levels (10 days IPTG removal). P-Akt (Thr_308_) protein levels remain unchanged in the presence of IPTG; during IPTG removal p-Akt (Thr_308_) protein levels increase considerably. GAPDH was used as loading control. Protein size markers are shown on the left of the figure. (**B**) Normalized (to GAPDH) expression levels of FAK, p-FAK (Tyr_397_), and CR in MSTO-shCALB2-IPTG treated for nine days (+IPTG) and after IPTG removal (-IPTG) for 10 days suggest a biological link of both proteins with CR levels. (**C**) Cell proliferation/viability measured with the MTT assay after treatment of MSTO-wt and MSTO-shCALB2-IPTG cells with the FAK inhibitor VS-6063 for 72 h. (**D)** MTT signals (proliferation/viability) measured after 72 h of treatment with different concentrations of Cis-Pt ranging from 0.625 µM to 5 µM in MSTO-wt, MSTO-CR and MSTO-shCALB2-IPTG (induced) cells (*n* = 4 independent experiments; * *p* ≤ 0.05, ** *p* ≤ 0.01, *** *p* ≤ 0.001).

Since altered FAK levels had been associated with increased tumor malignancy including tumor cell migration and invasiveness in cancer including MM [28,29] and moreover since we had discovered a link between CR and FAK [24], we investigated how reversible CR downregulation affected p-FAK (Tyr_397_) and FAK levels (Figure 3A). In the same samples we also determined levels of p-AKT (Thr_308_), because CR had been shown to affect PI3K/AKT signaling in MM cells before [26]. The initial IPTG-induced decrease of CR in MSTO-211H cells reaching its maximum of decrease at day 9, was essentially paralleled (with a delay of approximately three days) by a decrease in total FAK levels (Figure 3B). During the recovery period (IPTG removal for 10 days) restoring of CR levels to the ones before IPTG treatment also augmented expression levels of total FAK, and more prominently increased p-FAK (Tyr_397_) levels. The rather parallel changes of the three proteins, especially during recovery of CR expression are indicative of a positive correlation, possibly hinting towards a common regulation or even a causal relationship between CR and p-FAK/FAK levels. Qualitatively identical results were obtained in ZL55 cells; IPTG treatment for 11 days led to a decrease in p-FAK (Tyr_397_) levels, followed by a strong rise of p-FAK (Tyr_397_) after IPTG removal (Figure S1B).

As we had observed previously that CR overexpression in MSTO-211H cells resulted in an increased resistance towards the FAK inhibitor VS-6063 [24], consistent with increased levels of FAK and p-FAK (Tyr_397_) in these cells, we expected the opposite to occur in CR-depleted MSTO-211H cells. CR downregulation in MSTO-shCALB2-IPTG (induced) cells increased the sensitivity towards the FAK inhibitor, most evident at lower inhibitor concentrations of 2.5 and 5 µM (Figure 3C). This increased sensitivity is most likely a consequence of a decrease in the FAK signaling pathway activity caused by CR downregulation. Thus, CR downregulation in combination with FAK inhibitor treatment enhanced the efficacy of the FAK inhibitor to decrease cell viability. At higher VS-6063 concentrations (7.5 and 10 µM), cell viability was not affected by supplementary CR downregulation.

### 2.5. CR-Expressing Cells Display Higher Cis-Pt Resistance Possibly Mediated by Increased FAK and AKT Signaling

MM cells including MSTO-CR cells show increased activity of the FAK signaling pathway evidenced by higher p-FAK (Tyr_397_) levels compared to MSTO-wt cells, leading to an increased survival of CR-expressing cells in the presence of VS-6063 [24], while the inverse hold true for MSTO-CALB2-IPTG (induced) cells (Figure 3C). FAK is situated upstream of PI3K/AKT recruiting p85, the regulatory subunit of PI3K to phosphorylated FAK (Tyr_397_). The subsequent increase in PI3K activity leads to AKT pathway activation [30], and ultimately to an AKT-mediated evasion of apoptosis [25], thus enhancing cell survival. Activation of both FAK and PI3K/AKT signaling pathways are known to mediate chemoresistance in other malignancies [9,11]. Thus, we hypothesized that CR might exert also a protective effect towards Cis-Pt toxicity possibly via the same mechanisms, i.e., by activating FAK and subsequently PI3K/AKT. This was tested in MSTO-CR cells in comparison to the parental cells with respect to sensitivity to Cis-Pt. At 72 h post-treatment viability of MSTO-CR cells was higher than in MSTO-wt cells at the two lower Cis-Pt concentrations (0.625 and 1.25 µM) tested (Figure 3D). At the higher concentrations (2.5. and 5 µM) viability was decreased by 70–80% irrespective of CR overexpression, indicative of an overpowering effect of Cis-Pt. Additionally, p-AKT (Thr_308_) protein levels analyzed after IPTG induction and IPTG removal showed no changes at the protein levels in IPTG-treated cells; however, p-AKT (Thr_308_) levels were increased after IPTG removal and subsequent CR re-expression in the cells (Figure 3A), suggesting that the increased chemoresistance observed in CR-overexpressing MSTO-211H cells towards Cis-Pt might involve the activation of FAK/AKT downstream survival pathways. As this protective effect disappeared at high concentrations of Cis-Pt probably other, yet unknown pro-survival mechanisms, might contribute in mediating this observed effect. A comparison of MTT signals between ZL55-CR and ZL55-wt revealed no difference with respect to Cis-Pt resistance at all concentrations tested (Figure S2A).

### 2.6. Downregulation of CR Enhances Cis-Pt Resistance and Activates the Wnt Signaling Pathway in MSTO-shCALB2-IPTG (Induced) Cells

**Figure 4:**
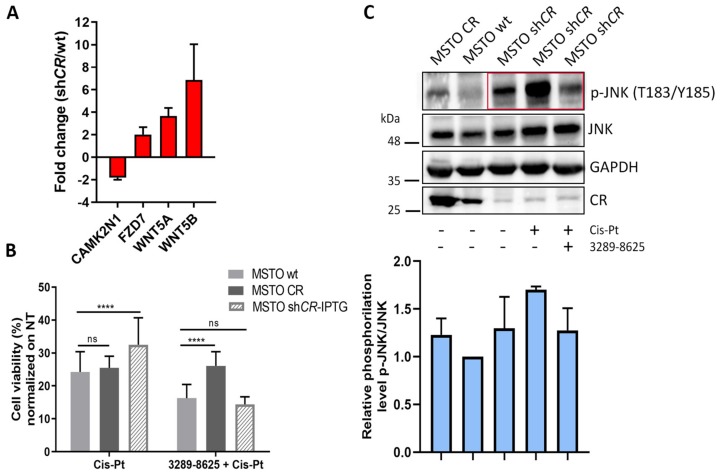
Effect of Cis-Pt and 3289-8625 on cell viability of MSTO-wt, MSTO-CR and MSTO-shCALB2-IPTG (induced) cells. (**A**) Relative fold-change of transcript expression determined by a PCR array in MSTO-shCALB2-IPTG (induced) cells compared to MSTO-wt cells. In CR-depleted cells note upregulation of gene transcripts related with the Wnt-signaling pathway (*FZD7*, *WNT5A* and *WNT5B*) and a decrease in the CaMKII inhibitor *CAMK2N1*. (**B**) MTT signals of MSTO-wt, MSTO-CR and shCALB2-IPTG (induced) cells after treatment (72 h) with Cis-Pt alone (left bars) or the combination (right bars) of the Wnt-pathway inhibitor 3289-8625 for 24 h (100 µM), followed by additional 48 h in the presence of Cis-Pt (2.5 µM; *n* = 5 independent experiments; asterisks represent **** *p* ≤ 0.0001). (**C**) Upper part: Representative Western blot signals of p-JNK (Thr_183_/Tyr_185_), JNK, and CR levels in untreated (CR-overexpressing) MSTO-CR, control MSTO-wt, and CR-depleted MSTO-shCALB2-IPTG (induced) cells, in MSTO-shCALB2-IPTG (induced) cells treated with Cis-Pt (5 µM) for 72 h alone, or pre-treated for 24 h with the Wnt inhibitor 3289-8625 (100 µM) followed by 48 h treatment of Cis-Pt (5 µM). The most pertinent signals for p-JNK (Thr_183_/Tyr_185_) are boxed in red. GAPDH was used as loading control. Lower part: Quantification of the relative JNK phosphorylation levels (ratio p-JNK/JNK normalized to GAPDH; ratio for MSTO-wt signals defined as 1.0) is shown (*n* = 3 independent experiments; mean ± SD). In CR-depleted (MSTO-shCR) cells, Cis-Pt treatment increases p-JNK (Thr_183_/Tyr_185_) (upper panel in **C**) and the ratio p-JNK/JNK, an effect that is reversed by the Wnt signaling inhibitor 3289-8625.

The few surviving MSTO-211H cells transduced with sh*CALB2* and subjected to IPTG-induced CR downregulation (Figure 1B) were exposed to the same range of Cis-Pt concentrations (0.625–5 µM) for 72 h. Unexpectedly, cells with decreased CR levels were also more resistant to Cis-Pt at all concentrations tested when compared to MSTO-wt cells. This was also the case at the higher Cis-Pt concentrations, where increased CR levels were ineffective in conferring cytotoxicity resistance (Figure 3D). The same held true for ZL55-shCALB2-IPTG (induced) cells, but only in cells exposed to the highest (5 µM) Cis-Pt concentration tested, when compared to ZL55-wt cells exposed to the same Cis-Pt concentration (Figure S2A). This might indicate that the Cis-Pt-mediated resistance was less pronounced in ZL55 cells and possibly not mediated by the same mechanism (see below). As p-AKT (Thr_308_) levels were unchanged and p-FAK (Tyr_397_) levels were decreased after CR downregulation in MSTO-211H cells (Figure 3A), both pathways were considered as unlikely contributors implicated in putative mechanisms mediating this protective effect. To further elucidate the potential mechanism implicated in the Cis-Pt resistance, MSTO-shCALB2-IPTG (induced) cells were analyzed using a specific PCR pathway array and compared to MSTO-wt cells. Transcript levels of several genes related to the Wnt-signaling pathway were differentially affected. Significant changes at the mRNA level (Figure 4A) included *WNT5A* and *WNT5B* transcripts (upregulated three- and six-fold, respectively), which encode non-canonical Wnt family members. Recent evidence links Wnt signaling to tumorigenesis in several cancer types and also in the development of cancer drug resistance [31]. Increased signaling due to upregulation of Wnt ligands, specifically Wnt5a, contributes to poor clinical outcome and increased drug resistance, e.g., in ovarian carcinoma and lung cancer [16,32], and also in MM cells [33]. *FZD7* mRNA levels were also increased; overexpression of different Frizzled (FZD) receptors have been also reported to over-activate the Wnt signaling pathway in a variety of cancers [34]. Ca^2+^/calmodulin-dependent protein kinase II inhibitor 1 transcripts (*CAMK2N1*), encoding a peptide composed of 78 amino acids, was downregulated two-fold in the CR-depleted cells. CaMK2N1 functions as a potent and specific inhibitor of calmodulin-dependent protein kinase II (CaMKII). CaMKII has recently emerged as a key protein in modulating cell proliferation, cell cycle, invasion/metastasis and therapy resistance in a variety of malignant diseases such as lung, breast, prostate and colon cancer. In addition, Wnt5a signals through the non-canonical pathway and stimulates intracellular Ca^2+^ release and activation of PKC and CaMKII [35,36]. In view of the above findings, we thus hypothesized that both canonical and non-canonical Wnt signaling pathways might play a regulatory role in the chemoresistance of MSTO-shCALB2-IPTG (induced) cells.

### 2.7. Inhibition of Wnt Signaling Re-Sensitizes Resistant MSTO-shCALB2-IPTG (Induced) Cells to Cis-Pt

With the aim of confirming that the chemoresistance observed in MSTO-shCALB2-IPTG (induced) cells was mediated via activation of the Wnt signaling pathway, cells were treated with the Wnt inhibitor 3289-8625, a cell-permeable aminobenzanilide compound that inhibits Dishevelled-2 (DVL2), an essential protein of the Wnt signaling pathway. DVL2 uses its PDZ domain to transduce the Wnt signals from the membrane receptor FZD to downstream components [37]. Cells were treated initially with 3289-8625 for at least 24 h and then exposed to Cis-Pt (2.5 µM) for an additional 48–72 h. In MSTO-wt cells, the Cis-Pt-induced decrease in viability was further potentiated by inhibition of DVL2. In Cis-Pt treated MSTO-CR cells, the inhibitor 3289-8625 had no effect on cell viability. However, in CR-depleted MSTO-shCALB2-IPTG (induced) cells, which initially showed higher resistance to Cis-Pt, DVL2 inhibition completely abrogated the protective effect and the low-CR cells were as sensitive as MSTO-wt cells (Figure 4B). A similar effect of DVL2 inhibition on increasing Cis-Pt sensitivity had been observed previously in lung cancer cells [38]. In summary, mechanisms implicated in the protective effect against Cis-Pt toxicity were different in CR-overexpressing cells (increased FAK signaling) and in cells with decreased CR levels, i.e., increased Wnt signaling in MSTO-shCALB2-IPTG (induced) cells.

### 2.8. Cis-Pt Resistance in MSTO-shCALB2-IPTG Cells is Mediated via the Activation of the Non-Canonical Wnt/JNK Pathway

**Figure 5:**
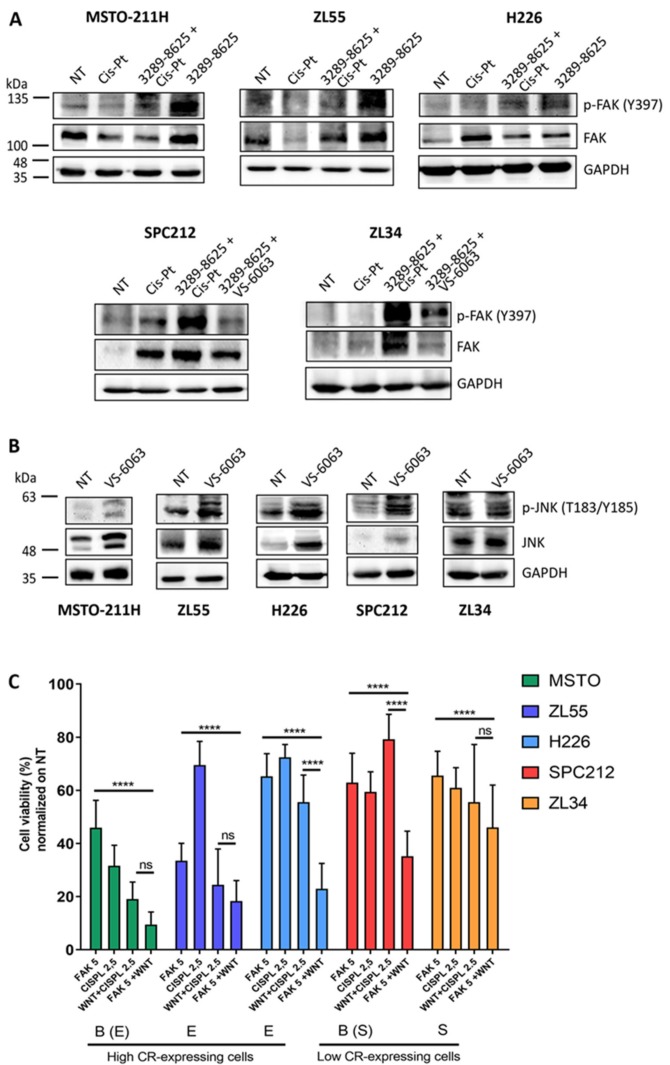
Western blot analysis of proteins implicated in FAK and Wnt signaling pathways in MM cells. (**A**) Western blot analysis of p-FAK (Tyr_397_) and total FAK after treatment with Cis-Pt (2.5 µM), 3289-8625 (100 µM) or the combination of 3289-8625 + Cis-Pt and 3289-8625 + VS-6063. GAPDH was used as loading control. (**B**) Levels of p-JNK (Thr_183_/Tyr_185_) and total JNK in MM cells treated with VS-6063 (5 µM). GAPDH was used as loading control (*n* = 3 independent experiments). Quantification of p-FAK (Tyr_397_) and total FAK signals in (**A**) and p-JNK (Thr_183_/Tyr_185_) and total JNK signals in (**B**) is shown in Figure S3. (**C**) Cell viability of MM cells measured by the MTT assay after 72 h of treatment with the different compounds. B biphasic, E epithelioid, S sarcomatoid (*n* = 5 independent experiments; asterisks represent ***p ≤ 0.001 and ****p ≤ 0.0001).

To further confirm which of the Wnt signaling pathways was involved in mediating the Cis-Pt resistance, protein levels of JNK (c-Jun N-terminal kinase) and the activated form p-JNK (Thr_183_/Tyr_185_) were analyzed in MSTO-wt, MSTO-CR and low-CR MSTO-shCALB2-IPTG (induced) cells. Initially, control (non-treated) MSTO-shCALB2-IPTG (induced) cells with decreased CR levels showed an increase in p-JNK (Thr_183_/Tyr_185_) levels when compared with MSTO-wt and MSTO-CR cells (Figure 4C). After treatment with Cis-Pt (5 µM), p-JNK (Thr_183_/Tyr_185_) levels were even more increased, implying a further activation of the Wnt/JNK pathway in mediating the Cis-Pt resistance of the MSTO-shCALB2-IPTG (induced) cells. When pre-treated with the DVL2 inhibitor 3289-8625, levels of p-JNK (Thr_183_/Tyr_185_) were attenuated and similar to non-treated cells. Of note, unlike in MSTO-211H cells, where the Cis-Pt-induced resistance was abrogated by 3289-8625, the Cis-Pt-augmented resistance in ZL55 cells was unaffected by 3289-8625 (Figure S2B). In agreement, although levels of p-JNK (Thr_183_/Tyr_185_) were similarly (as in MSTO-CALB2-IPTG (induced) cells) increased after 11 days of IPTG induction in ZL55-shCALB2-IPTG (induced) cells (Figure S2C), treatment with Cis-Pt decreased levels p-JNK (Thr_183_/Tyr_185_) and moreover the additional treatment with 3289-8625 had no effect on p-JNK (Thr_183_/Tyr_185_) levels. This indicates that in CR-depleted ZL55 cells the activation of the non-canonical Wnt signaling way is probably not implicated in the Cis-Pt resistance mechanism (see also Figure 5A). As recently shown in ZL55 cells, Cis-Pt induces the generation of reactive oxygen species (ROS), which activates PKC-α leading to EGFR transactivation triggering the MAPK pathway evidenced by phosphorylation of ERK1/2 [39]. Thus, in this particular cell line other pro-survival mechanisms (likely including MAPK signaling) is involved in conferring Cis-Pt resistance, since inhibition of MAPK signaling at various levels (PKC-α, EGFR, or ERK1/2) increases Cis-Pt cytotoxicity.

### 2.9. Antagonistic Regulation of the FAK and Wnt Signaling Pathways in MM Cells

In view of the above findings, we investigated the possible interplay between the FAK and Wnt signaling pathways in different MM cell lines independently of CR expression levels or of modulation by CR. FAK is known to modulate Wnt in a complex and dynamic manner in different cell types working either upstream or downstream of Wnt signaling, and at times even synergistically [40]. For these experiments we used three MM cell lines derived from epithelioid (ZL55, H226) or biphasic (MSTO-211H with mostly epithelioid morphology and characteristics) MM, and two cell lines consisting completely (ZL34) or predominantly (SPC212) of cells with sarcomatoid morphology. In the presence of the Wnt inhibitor 3289-8625, levels of p-FAK (Tyr_397_) were increased in all cell lines (in SPC212 and ZL34 cells seen as the difference between Cis-Pt alone and Cis-Pt + 3289-8625) indicating universal activation of the FAK signaling pathway (Figure 5A). Unlike in the “sarcomatoid” lines SPC212 and ZL34, co-addition of Cis-Pt had no significant effect on p-FAK (Tyr_397_) levels in ZL55, H226 and MSTO-211H cells. Treatment with Cis-Pt alone did not reveal a congruent picture: a weak up-regulation in SPC212 cells, a small decrease in MSTO-211H and ZL55 cells and no obvious changes in the others. Conversely, treatment with the FAK inhibitor VS-6063 (5 µM) strongly increased p-JNK (Thr_183_/Tyr_185_) and total JNK levels in all cell lines except ZL34 (Figure 5B). Quantitative analyses of the normalized p-FAK, total FAK and p-JNK, total JNK signals are shown in Figure S3.

These results strongly indicated antagonistic regulation of these two pathways, i.e., FAK inhibition up-regulating Wnt signaling and vice versa in MM cells. Finally, we compared the effects of the clinically approved drug Cis-Pt alone, VS-6063 alone and the combinations 3289-8625 and Cis-Pt and 3289-8625 and VS-6063 using the MTT assay reporting the combined effects of cell proliferation and viability. All four treatments in all five MM cell lines resulted in lower MTT signals, the minimal decrease being around 20% in SPC212 cells (Figure 5C). The sensitivity towards the various treatments was generally higher in “epithelioid” cells (MSTO-211H, ZL55, H226) than in “sarcomatoid” (SPC212, ZL34) cells. In all MM cell lines, blocking both FAK and Wnt signaling pathways most strongly decreased MTT signals, although the combination of Cis-Pt and 3289-8625 was almost equally effective in the lines MSTO-211H, ZL55 and ZL34. The effect of the various drugs and combinations on cell proliferation (confluence) and cell morphology is shown in Figure S4.

In parallel, the effects of the identical treatments were tested in Met-5a cells, a SV40-immortalized mesothelial cell line and LP9/TERT-1 cells, considered as a model for reactive mesothelial cells (Figure S5). All treatments in both cell lines led to a decrease of the MTT signal in the order of 20%, in the case of the combination of Cis-Pt and 3289-8625 of 40%. Of importance, the combination of 3289-8625 and VS-6063 was not more toxic in non-transformed mesothelial cells than the approved drug Cis-Pt, indicating that blocking FAK and Wnt signaling pathways more strongly affected tumor cells than untransformed (normal) mesothelial cells.

## 3. Discussion

Cis-Pt-based chemotherapy is a commonly used anti-cancer treatment for MM; Cis-Pt induces apoptosis by suppressing DNA replication of cancer cells [41]. The efficacy of chemotherapeutic drugs depends not only on their capacity to induce DNA damage, but also on the cells’ detection systems and responsiveness to DNA damage [42]. Many tumors show (acquired) resistance to Cis-Pt, primarily by inactivating apoptotic factors and/or enhancing cell survival pathways [8]. Among the signaling pathways involved in enhancing Cis-Pt resistance, aberrant activation of PI3K/AKT, FAK and Wnt/β-catenin signaling were observed before [9,10,11]. FAK activity is directly linked to the pro-survival PI3K/AKT signaling axis through the recruitment of the regulatory subunit of PI3K [25], which in turn leads to AKT-mediated evasion of apoptosis. Since increases in CR expression in MM cells leads to increased p-FAK (Tyr397) levels [24], the higher Cis-Pt resistance observed in MSTO-CR cells might be directly associated with the hyper-activation of the FAK-PI3K/AKT survival pathway. Consistently, recovery of CR expression after IPTG removal in MSTO-shCALB2-IPTG (induced) cells resulted in a strong increase in p-AKT (Thr308) levels. In line with this finding, CR protects immortalized Met-5A mesothelial cells from acute asbestos-induced cytotoxicity also via the activation of the PI3K/AKT signaling pathway [26]. Unexpectedly, MSTO-shCALB2-IPTG (induced) cells were more resistant to Cis-Pt, a finding not explainable by the activation of the FAK-PI3K/AKT axis, since no changes in p-AKT (Tyr308) and even a decrease in p-FAK (Tyr397) levels were observed after CR downregulation. Thus, low-CR expressing cells with lower p-FAK (Tyr397) levels were more susceptible towards the FAK inhibitor VS-6063. This hinted towards other mechanisms implicated in Cis-Pt resistance in MSTO-shCALB2-IPTG (induced) cells.

A PCR array revealed an increase in Wnt signaling-related genes in MSTO-211H cells, where CR expression was downregulated. In addition to its role in development, tissue regeneration and homeostasis, dysregulated Wnt signaling plays a role in neoplastic properties, e.g., progression, invasion and resistance to apoptosis of MM cells [13,14,33] and moreover in the development of cancer drug resistance [31]. Increased signaling due to upregulation of Wnt ligands, specifically Wnt5a, contributes to poor clinical outcome and increased drug resistance, e.g., in ovarian carcinoma and lung cancer [16,32]. Additionally, MM cells express various Wnt family members including Wnt2b, Wnt3, Wnt4 and Wnt5a [13,14,33]; blocking of Wnt3a activity by secreted frizzled related peptide 4 (SFRP4) inhibits cell proliferation and migration of JU77 and ONE58 MM cells [43]. Wnt family members are secreted glycoproteins (at least 19 in humans) that transduce signals by binding to specific Frizzled (FZD) receptor complexes (reviewed in [44]). In particular, FZD7 activates Wnt/β-catenin signaling in hepatocellular carcinoma, colon cancer and TNBC (triple negative breast cancer) cells [45]. FZD7 also regulates Wnt/JNK signaling in colon cancer and is, therefore, capable to activate both, Wnt canonical and non-canonical signaling pathways [46]. Hyperactivation of JNK proteins leads to the activation of downstream pathways related to an increase of drug resistance leading to an escape of apoptosis [17]. mRNA levels of WNT5A, WNT5B and FZD7 were consistently augmented in MSTO-shCALB2-IPTG (induced) cells (Figure 4A). Wnt5a, a representative Wnt ligand of the non-canonical pathway and FZD7, a receptor for secreted WNT proteins, promote cancer cell metastasis, EMT and chemoresistance in several cancers [16,47]. Based on the dependence of its key mediator β-catenin, the Wnt signaling pathway is subdivided in a canonical (β-catenin-dependent) and a non-canonical β-catenin-independent) one [48]. Non-canonical Wnt pathways include the planar cell polarity (PCP) and the Wnt/calcium pathways [49]. In the latter, the ligand binding of Wnt receptor/coreceptor(s) leads to an increase in the intracellular calcium concentration [Ca^2+^] that activates CaMKII and calcineurin. CaMKII has recently emerged as a key protein in modulating cell proliferation, cell cycle, invasion/metastasis and therapy resistance in a variety of malignant diseases such as lung, breast, prostate, and colon cancer. In addition, Wnt5a signals through the non-canonical pathway and stimulates intracellular Ca^2+^ release and activation of PKC and CaMKII [35,36]. In our study, levels of CAMK2N1, a specific inhibitor of CAMKII, were decreased in MSTO-shCALB2-IPTG (induced) cells suggesting a likely major involvement of the non-canonical pathway in mediating the Cis-Pt chemoresistance. The PCP pathway involves Jun N-terminal kinase (JNK) activation [50] and in line with this, CR-depleted MSTO-211H cells showed an increase in p-JNK (Thr183/Tyr185) protein expression levels. The increase was further enhanced after Cis-Pt treatment of the cells, implicating an involvement of the Wnt/JNK pathway in mediating the increased Cis-Pt resistance. The JNK pathway plays a significant role in mediating induction of cell-cycle arrest and apoptosis in response to Cis-Pt [51]. Dishevelled (DVL) is an essential protein in the Wnt signaling pathways as well [52]. It makes use of its PDZ domain to transduce Wnt signals from the membrane receptor FZD to downstream components to initiate the signaling cascade. The compound 3289–8625 efficiently blocks Wnt signaling by directly binding the PDZ domain of DVL [37], re-sensitizing chemoresistant cells in several cancers to Cis-Pt, such as colon or lung cancer [38,53]. Accordingly, treatment of MSTO-shCALB2-IPTG (induced) cells with 3289–8625 completely abrogated their increased resistance to Cis-Pt and restored sensitivity as in MSTO-wt cells (Figure 4B).

Constitutive CR downregulation via lentiviral-mediated sh*CALB2* strongly decreases proliferation and strongly impairs survival of MM cells in vitro [27]. This was true, to a lesser extent, also with the IPTG-inducible system used in this study. However, the slower kinetics of the effects mediated by the IPTG-induced CR downregulation, likely more representative for a condition prevailing in vivo in comparison to directly injecting sh*CALB2*-infected MSTO-211H cells [24] allowed to investigate various signaling pathways affected by lowering CR expression levels. The first observable effects included a decreased migratory and invasive phenotype in vitro. The inverse experiment, i.e., overexpression of CR in MSTO-211H MM cells, was previously shown to increase migration and invasion in vitro [24]. In line with both findings, constitutive CR downregulation in MSTO-211H cells pretreated (transduced) with sh*CALB2*-producing lentivirus, followed by injection of these low-CR cells in a mouse xenograft model in vivo resulted in a significant decrease of tumor growth in comparison to the parental MSTO-211H cells [24]. Here, we demonstrated that slower CR downregulation in vitro resulted in the survival of a subpopulation of Cis-Pt-resistant cells, most likely resulting from upregulation of (survival) genes of the non-canonical Wnt signaling pathway.

There is increasing evidence of a crosstalk between the FAK and Wnt signaling pathways. FAK is required for the proper regulation of Wnt/β-catenin signaling during early vertebrate development. In breast and pancreatic cancer cells, FAK is assumed to act up-stream of the Wnt/β-catenin pathway [40], while in other systems, FAK acts downstream of Wnt promoting AKT/mTOR activation in vivo and leading to increased intestinal cell proliferation and tumorigenesis [54]. In HEK cells, Wnt and FAK were shown to work synergistically to regulate expression of downstream target genes. A plethora of FAK/Wnt interactions are achievable, suggesting a complex interaction [40] and as shown in our study resulting in antagonistic regulation of both pathways. To add even more complexity in MM cells, CR levels seem to modulate or to be modulated by both pathways. Although a recent study reported that MM cells lacking merlin were more sensitive to the FAK inhibitor VS-4718 in vitro and in tumor xenograft models in vivo [55], a phase II clinical trial investigating the effect of the FAK inhibitor VS-6063 alone (NCT01870609) failed in showing any benefit due to lack of efficacy in patients (for other ongoing or planned trials with FAK inhibitors including VS-6063, see https://clinicaltrials.gov/ct2/home). Thus, targeting several pathways (FAK and Wnt), possibly in combination with CR downregulation should be considered as a prospective strategy for the treatment of MM. Since currently no small molecule inhibitors of CR function/expression are available, the concomitant targeting of druggable signaling pathways appears more promising.

## 4. Materials and Methods

### 4.1. Cell Culture

The human MM cell lines MSTO-211H and H226, in addition to MeT-5A, HeLa and HEK293T cells were obtained from the American Type Cell Collection (ATCC, Rockville, MD, USA). The MM cell lines ZL55, SPC212 and ZL34, were obtained from the University Hospital of Zurich (Zurich, Switzerland) [56], and the LP9/TERT-1 from the laboratory of Dr. James Rheinwald (Dana Farber Cancer Research Institute, Boston, MA, USA). HeLa and HEK293T cells were maintained in DMEM supplemented with 10% FBS (Gibco, Basel, Switzerland) and 1% penicillin/streptomycin solution (1% PS; Gibco). The rest of the cell lines, as well as the parental MSTO-211H cells and derived genetically modified lines were cultured in RPMI-1640 (Sigma-Aldrich, Buchs, Switzerland) containing 10% FBS (Gibco) supplemented with 2.5 μg/mL amphotericin B (Corning, Oneonta, NY, USA). All cells were maintained at 37 °C in a humidified 5% CO_2_ atmosphere.

### 4.2. pLKO-puro-IPTG-3xLacO Inducible shRNAs System Plasmids

The plasmid TurboGFP-C1 (#54728) was obtained from Addgene and the coding sequence for TurboGFP was inserted into the pLVTHM vector (Addgene; https://www.addgene.org/, #12247). For this, the GFP cassette in pLVTHM was replaced with the cDNA coding for TurboGFP. The required fragment was synthesized by PCR using the primers PmeI-ATG GAG AGC GAC GAG AGC GGC and SpeI- TTA TCT AGA TCC GGT GGA TCC. The amplicon was digested with PmeI and SpeI and inserted into the unique sites of the pLVTHM vector to produce the final TurboGFP plasmid. The pLKO-puro-IPTG-3xLacO inducible sh*RNA* vectors targeting human *CALB2* and *TurboGFP* were purchased from Sigma-Aldrich.

### 4.3. Lentiviral (LV) Production and Titration

For CR overexpression, the plasmid pLV-CALB2 was used as shown previously [57]. Lentivirus particles were produced as described before [58]. Briefly, HEK293T cells were co-transfected by the CaPO_4_ method with 3 µg of the envelope plasmid pMD2.G-VSVG (Addgene plasmid #12259), 8 µg of the packaging plasmid psPAX2 (Addgene plasmid #12260) and 10 µg of the transfer plasmid (TurboGFP; pLKO-puro-IPTG-3xLacO shCALB2 and pLKO-puro-IPTG-3xLacO shTurboGFP). Lentivirus in the supernatant of HEK293T cells were harvested 48 and 72 h after transfection. The supernatant was filtered (0.45 µM) and resuspended in DMEM containing 10% FBS and 1% PS solution. For titration, lentiviral particles at dilutions of 10^−3^ to 10^−7^ were used to infect HeLa cells in six-well plates (100,000 cells/well). The medium was replaced after 48h with a selection medium containing 2 µg/mL puromycin (Sigma-Aldrich). At day 12 post-infection, cells were stained with crystal violet and colonies counted to determine the lentiviral titer.

### 4.4. Chemicals and Reagents

The FAK inhibitor Defactinib (VS-6063, PF-04554878) was obtained from Selleckchem (Houston, TX, USA). Cisplatin (Cis-Pt) and dimethyl sulfoxide (DMSO) were purchased from Sigma-Aldrich. The compound DVL PDZ-domain inhibitor II 3289-8625 was obtained from Merck, Schaffhausen, Switzerland (catalog number 322338). All compounds were dissolved in DMSO. Medium containing 0.5% DMSO was used as control for the experiments. A concentration of 1 mM IPTG (Isopropyl β-d-1-thiogalactopyranoside; PanReac AppliChem, Axonlab, Baden-Dättwil, Switzerland) was used for the induction of CR downregulation; the medium containing IPTG was replaced every three days.

### 4.5. Validation of the pLKO-puro-IPTG-3xLacO shTurboGFP and pLKO-puro-IPTG-3xLacO shCALB2 Inducible Constructs

MSTO-211H cells were transduced with a lentiviral vector containing the TurboGFP expression plasmid (at MOI 10) resulting in constitutive TurboGFP expression. The stably integrated pLKO-puro-IPTG-3xLacO shTurboGFP (used as control) and the pLKO-puro-IPTG-3xLacO shCALB2 inducible constructs were used to induce either *TurboGFP* or *Calb2* shRNA expression after IPTG addition to the medium in MSTO-211H cells stably expressing TurboGFP. In order to validate the control inducible system pLKO-puro-IPTG-3xLacO sh*TurboGFP*, cells were treated with IPTG and the green fluorescence was tracked in real-time using the Incucyte™ Live-cell Imaging System (Essen Bioscience Inc., Ann Arbor, MI, USA). To test for CR downregulation in the pLKO-puro-IPTG-3xLacO sh*CALB2* system, protein extracts from IPTG-treated cells were taken from day 1 to 9 and at days 1–12 after IPTG removal to check for reversibility of the effect. Protein extracts were subjected to Western blot analyses. Growth curves were determined by the Incucyte™ system and cell viability was measured with the MTT assay performed after nine days of IPTG induction. For the following experiments nine days of treatment with IPTG was used as the reference time point for sh*CALB2*-IPTG induced cells.

### 4.6. Western Blotting

Cells were collected using a cell scraper and protein extracts were obtained by using standard RIPA buffer (50 mM Tris, 150 mM NaCl, 0.1% sodium dodecyl sulfate (SDS), 0.5% sodium deoxycholate, 1% Triton X-100, pH 7.4) containing a protease inhibitor cocktail (Quartett GmbH, Berlin, Germany), and 1 mM sodium orthovanadate (Na_3_VO_4_; Sigma-Aldrich). Protein concentrations were determined using the BCA assay (BC Assay Protein Quantitation Kit, Uptima, Interchim, Montluçon, France) and equal amounts of protein (40 μg) were separated by SDS-PAGE (10%) and transferred onto nitrocellulose membranes [27]. Membranes were blocked with 5% BSA in TBS-Tween 20 for 1 h and incubated overnight at 4 °C with primary antibodies diluted in 2% BSA: rabbit polyclonal anti-calretinin (1:10,000; CR 7699/4, Swant, Marly, Switzerland), rabbit polyclonal anti-GAPDH (1:5000; Sigma); and the following antibodies from Cell Signaling Technology (Danvers, MA, USA): rabbit polyclonal anti-FAK (#3285, 1:1000), rabbit polyclonal anti-p-FAK-Tyr_397_ (#3283, 1:1000), rabbit polyclonal anti p-Akt-Thr_308_ (#4056; 1:1000), rabbit polyclonal anti-p-SAPK/JNK (Thr_183_/Tyr_185_) (#9251, 1:1000) and rabbit polyclonal anti-SAPK/JNK (#9252, 1:1000). The following day membranes were incubated with secondary goat anti-rabbit (HRP)-labeled antibodies (Sigma-Aldrich) at a dilution of 1:10,000. Signals were detected as described in [27].

### 4.7. Migration and Invasion Assays

Migration and invasion of MM cells was assessed as described before [24]. Briefly, MSTO-wt and MSTO-shCALB2-IPTG (induced) cells were grown to confluence in 96-well ImageLock plates (Essen Bioscience) pre-coated with a thin layer of 0.1 mg/mL Matrigel Basement Membrane Matrix (Corning, Oneonta, NY, USA, Cat. No.354234). A scratch of about 1 mm was created using the 96 well-plate woundmaker tool (Essen Bioscience) as described by the manufacturer. In the case of the invasion assay, 50 µL of Matrigel (0.6 mg/mL) was added to each well after the scratch. After 30 min incubation, 100 µL of medium was added on top and plates were scanned at a 2 h-frequency using the Incucyte™ system (Essen Bioscience). Images were evaluated with the IncuCyte™ software system (version 2011A, Essen Bioscience).

### 4.8. Cell Treatments and MTT Assays

MTT assays were used to assess the sensitivity of the cells to the FAK inhibitor VS-6063, cisplatin (Cis-Pt), and to the DVL inhibitor 3289-8625 in combination with Cis-Pt. For the FAK inhibitor assay, 3 × 10^3^ cells/well were seeded in 96-well plates (TPP Techno Plastic Products AG, Trasadingen, Switzerland) and grown for 48 h. Then the compound VS-6063 was added in a concentration range from 2.5 to 10 µM and after 72 h the MTT was added to each well at a final concentration of 0.5 mg/mL. To test for Cis-Pt sensitivity, 2.5 × 10^3^ cells/well were seeded in 96-well plates and treated with various concentrations of Cis-Pt (0.625, 1.25, 2.5, 5 µM). The MTT assay was performed 72 h after starting with the treatment. For experiments with 3289−8625 (100 µM), the compound was added to 2 × 10^3^ cells/well for 24 h, after which cells were treated with Cis-Pt (2.5 µM) for an additional 48 h. For the combination of VS-6063 and 3289-8625, cells were treated with both inhibitors at the same time applying the conditions mentioned above. For all experiments, the absorbance at 590 nm was measured using a microplate reader (Victor X3 2030 multilabel reader, Perkin-Elmer, Waltham, MA, USA). The MTT signal for control cells was defined as 100%. Additionally, protein extracts of cells treated with Cis-Pt, and/or with the different inhibitors were subjected to Western blotting.

### 4.9. PCR Array

Total RNA from MSTO-wt and MSTO-shCALB2-IPTG (induced) cells was isolated using the RNeasy mini kit (Qiagen, Hombrechtikon Switzerland) and then reverse-transcribed into single stranded cDNA with the RT^2^ First Strand Kit (Qiagen, Hilden, Germany) following the manufacturer’s instructions. A RT^2^ profiler PCR array specific for the EMT pathway (PAHS-090ZR-12, Qiagen) was performed to analyze differences in gene expression of the MM cells. Quantitative real-time PCR (RT-qPCR) was performed using a DNA thermal cycler (Corbett Rotor gene 6000, QIAGEN Instruments AG, Hombrechtikon, Switzerland). The following thermal profile was applied: one cycle at 95 °C for 10 min, 40 cycles at 95 °C for 15 s, and 60 °C for 30 s. Differences in fold expression were calculated according to the 2^−ΔΔC*t*^ method [59].

### 4.10. Statistical Analysis

A two way-ANOVA was used to analyze the MTT assay data (factors: CR levels and treatment); a Sidak’s multiple comparison was used as post-hoc test. MTT results from three to six independent experiments were pooled together; each sample was measured in triplicates. For the MTT assay of samples subjected to IPTG treatment, an unpaired t-test (two-tailed) was used. Mean and standard deviation are shown in the figures. GraphPad Prism software (8.0.0 version, GraphPad Software Inc., San Diego, CA, USA) was used to calculate the statistical significance of all the experiments. Differences with p-values of less than 0.05 were considered significant.

## 5. Conclusions

The lack of “single driver mutations” in MM presents an even higher challenge for the development of molecular-targeted therapies, as several mutations and several dysregulated pathways characterize this disease, highlighting a need for a better understanding of MM biology [60]. Even though FAK inhibitors have demonstrated a high anti-tumor efficacy in some in vitro and mice xenograft models [55,61], the use of these inhibitors has shown lack of efficacy in early clinical trials on patients [62], suggesting an involvement of other yet unidentified pathways in favoring tumorigenesis. “No man is an island”, and similarly no pathway can be modulated without affecting others [19]. Several signaling cascades influence Wnt signaling dynamics; in addition there is increasing evidence of a crosstalk between the FAK and the Wnt signaling pathways [40]. Such a crosstalk between different signaling pathways, together with the unpredictable and dynamic cell derangement response to overcome the toxic effect of treatments, e.g., alterations in drug metabolism, changes of signaling pathways, modification of apoptotic signaling or interference with cell replication [63], all limit the efficacy of therapeutic approaches with agents targeting single entities (molecules, pathways). Combinations of therapies directed at different targets instead towards single factors are being explored and start to demonstrate stronger effects [64]. In view of our findings, co-targeting of CR, a direct activator of the FAK signaling pathway, in combination with other key signaling pathways, such as Wnt, may be considered as a potential new strategy aimed at improving the current therapeutic landscape of MM.

## Supplementary Materials

Supplementary materials can be found at https://www.mdpi.com/1422-0067/20/21/5391/s1.

## Abbreviations

CR	calretinin
FAK	focal adhesion kinase
MM	malignant mesothelioma
EMT	epithelial-to-mesenchymal transition
DVL	Dishevelled
