# Supplementary material for: Modulation of Calretinin Expression in Human Mesothelioma Cells Reveals the Implication of the FAK and Wnt Signaling Pathways in Conferring Chemoresistance towards Cisplatin
Source: Int J Mol Sci. 2019 Oct 29;20(21):5391. doi: 10.3390/ijms20215391 (PMC6873109; doi:10.3390/ijms20215391)
Supplement: Supplementary file 1 [file ijms-20-05391-s001.pdf]

Article

Modulation of Calretinin Expression in Human Mesothelioma Cells Reveals the Implication of the FAK and Wnt Signaling Pathways in Conferring Chemoresistance towards Cisplatin

Janine Wörthmüller, Valérie Salicio, Anne Oberson, Walter Blum and Beat Schwaller

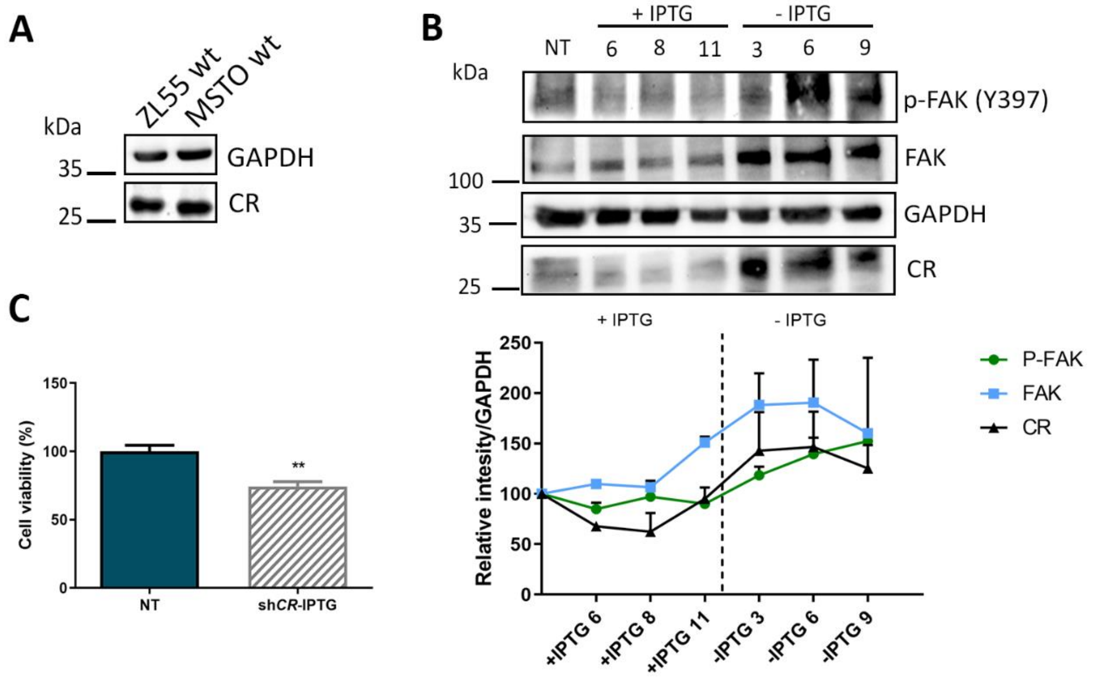

Figure S1. Analysis of different protein levels and viability on ZL55 cells before and after sh*CR*-IPTG induction. (**A**) Western blot analyses of CR levels in the parental (wt) ZL55 and MSTO-211H cell lines. GAPDH was used as loading control. Protein size markers are shown in the figure. (**B**) Western blot analyses of CR, p-FAK (Tyr_{397}) and total FAK levels of extracts from ZL55-shCALB2-IPTG cells induced with IPTG for 11 days (+IPTG) and after IPTG removal (-IPTG) up to 9 days. GAPDH was used as loading control. In the lower panel, normalized values (to GAPDH) of Western blot signals of the 3 proteins are shown (n = 3 independent experiments; mean + SD). Of note, signals for both, p-FAK (Tyr_{397}) and total FAK change within the time course of the experiment. (**C**) Cell number/viability, determined by an MTT assay shows a decrease in the number of viable ZL55-shCALB2-IPTG cells by 30% (**$p \leq 0.05$) after 8 days of IPTG treatment compared with non-treated (NT) cells.

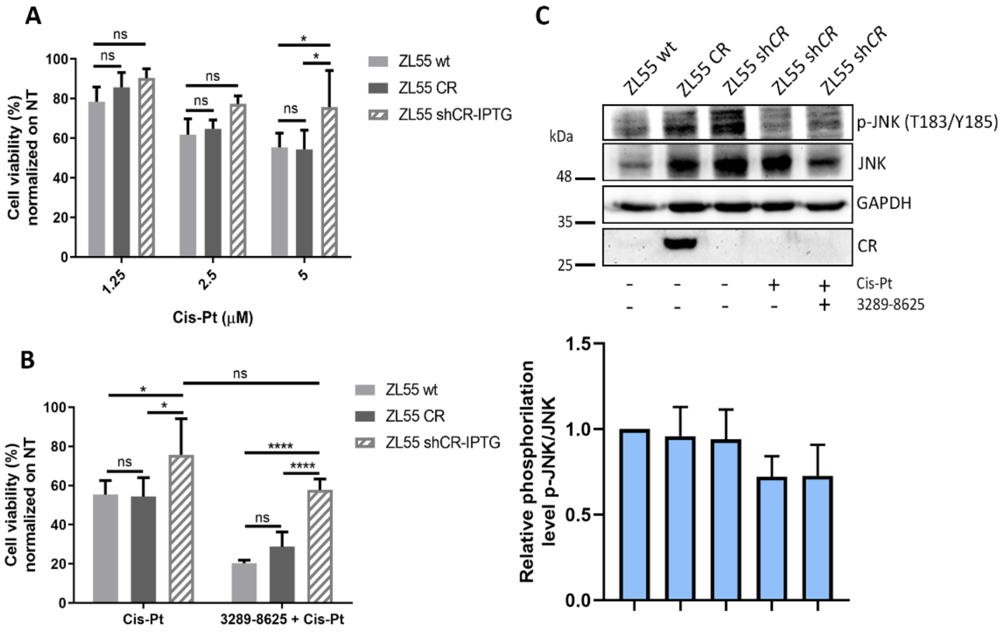

Figure S2. Effect of Cis-Pt and the Wnt inhibitor 3289-8625 on cell viability and on expression levels of different proteins of ZL55-wt, ZL55-CR and ZL55-shCALB2-IPTG (induced) cells. (**A**) MTT signals measured after 72 h of treatment with different concentrations of Cis-Pt ranging from 1.25 μM to 5 μM in ZL55-wt, ZL55-CR and ZL55-shCALB2-IPTG (induced) cells ($n = 3$ independent experiments; * $p \leq 0.05$). (**B**) MTT signals of ZL55-wt, ZL55-CR and ZL55-shCALB2-IPTG (induced) cells after 24 h of treatment with 3289–8625 (100 μM), followed by additional 48 h in the presence of Cis-Pt (5 μM; $n = 3$ independent experiments; asterisks represent * $p \leq 0.05$, and **** $p \leq 0.0001$). (**C**) Upper part: Representative Western blot signals of CR, JNK, and p-JNK (Thr_{183}/Tyr_{185}) in untreated (CR-overexpressing) MSTO-CR, control MSTO-wt and CR-depleted ZL55-shCALB2-IPTG (induced) cells, in ZL55-shCALB2-IPTG (induced) cells treated with Cis-Pt (5 μM) for 72 h alone, or pre-treated for 24 h with 3289–8625 (100 μM) followed by 48 h treatment of Cis-Pt (5 μM). GAPDH was used as loading control. Protein size markers are shown in the figure. Lower part: Quantification of the relative JNK phosphorylation levels (ratio p-JNK/JNK normalized to GAPDH; ratio for ZL55-wt cells defined as 1.0) is shown ($n = 3$ independent experiments; mean + SD).

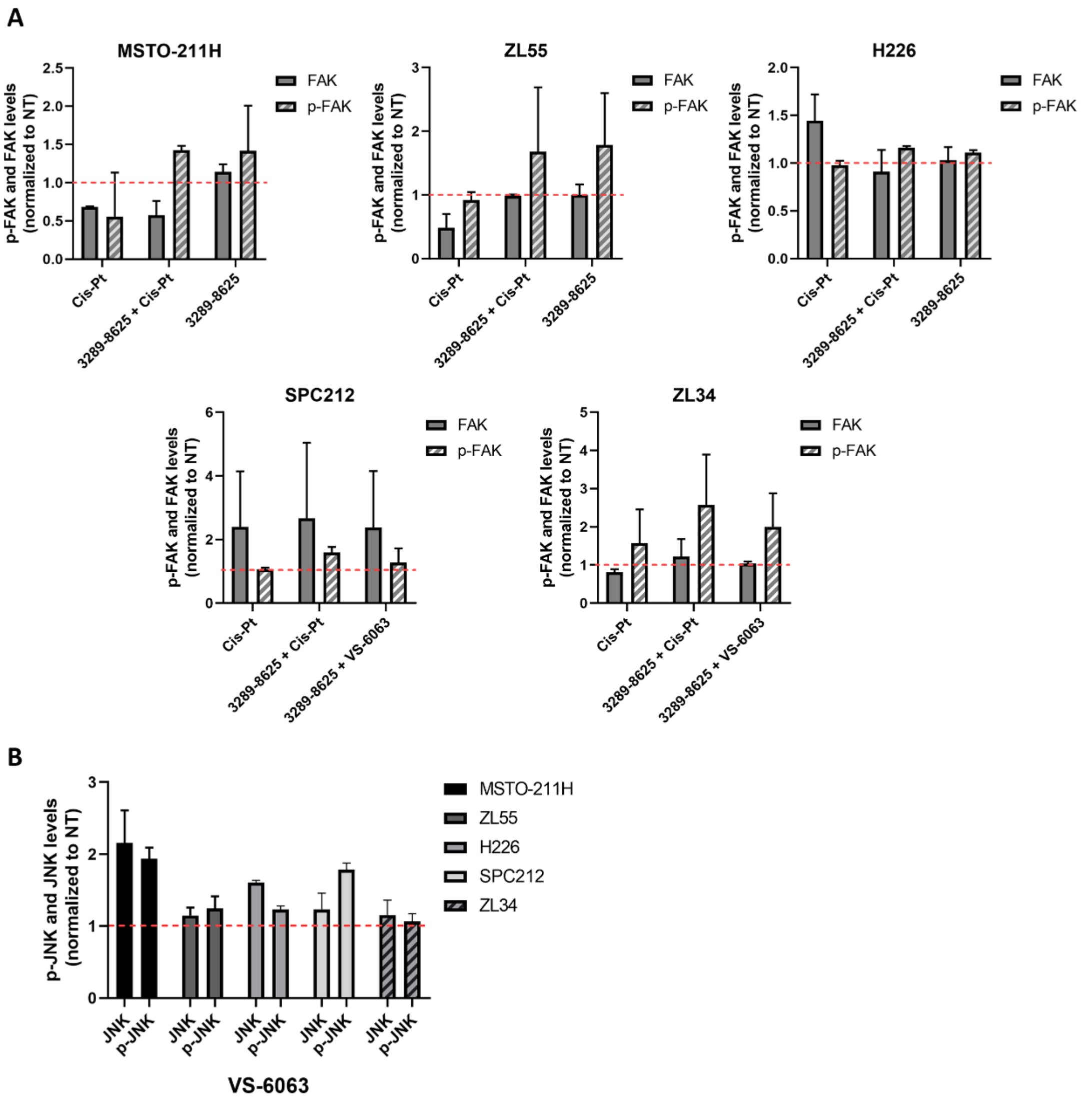

Figure S3. Quantification of Western blot data presented in Figure. 5. (**A**) For each cell line and each experiment the normalized signals (to GAPDH) of untreated (NT) cells was defined as 1.0 and the changes caused by the various treatments are shown in the bar graphs. Since treatments affected both, p-FAK (Tyr$_{397}$) and total FAK signals, the changes for both signals are shown separately (*n* = 3 independent experiments for all cell lines and treatments). Of note, higher values for normalized p-FAK (Tyr$_{397}$) and total FAK signals in a particular cell line (compared to other cell lines) do not indicate "stronger" pathway activation; values >1 are indicative of pathway activation. In some cell lines, mostly p-FAK (Tyr$_{397}$) signals are increased (MSTO-211H, ZL55, ZL34), in others levels of total FAK (SPC212) or both (H226). **(B)** Quantification of the effect caused by treatment with the FAK inhibitor VS-6063. Analyses were performed as described in (**A**), i.e. normalized to untreated (NT) cells. Since treatment changed signals for both, p-JNK (Thr$_{183}$/Tyr$_{185}$) and total JNK, signal changes for both are shown separately. The VS-6063-induced increase in both signals is indicative of upregulation also of JNK protein levels in all MM cell lines except ZL34 (n = 3 independent experiments for all cell lines treated with VS-6063).

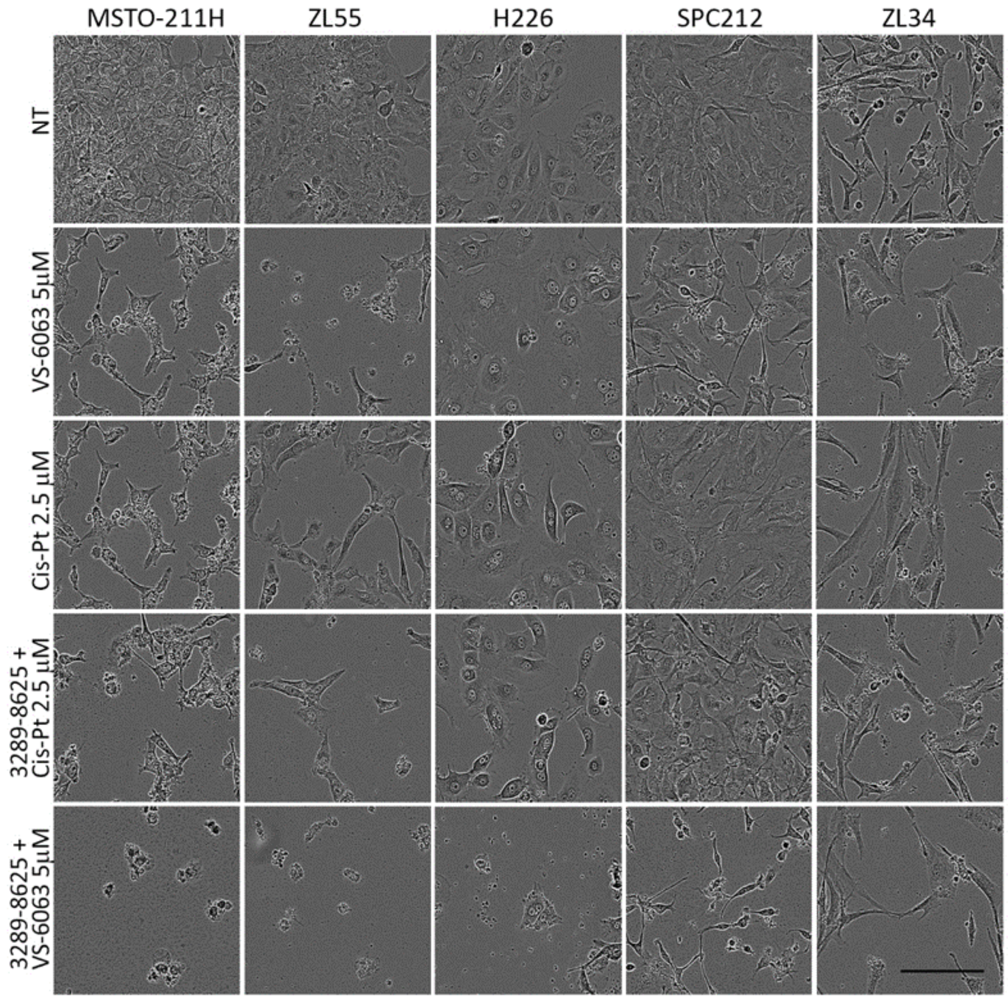

Figure S4. Effect of the FAK inhibitor VS-6063, Cis-Pt, a combination of the Wnt inhibitor 3289-8625 + Cis-Pt or 3289-8625 + VS-6063 on cell proliferation (confluence) and morphology. Brightfield images of the different MM cell lines were taken 72 h after the start of treatments. Scale bar: 400 μm.

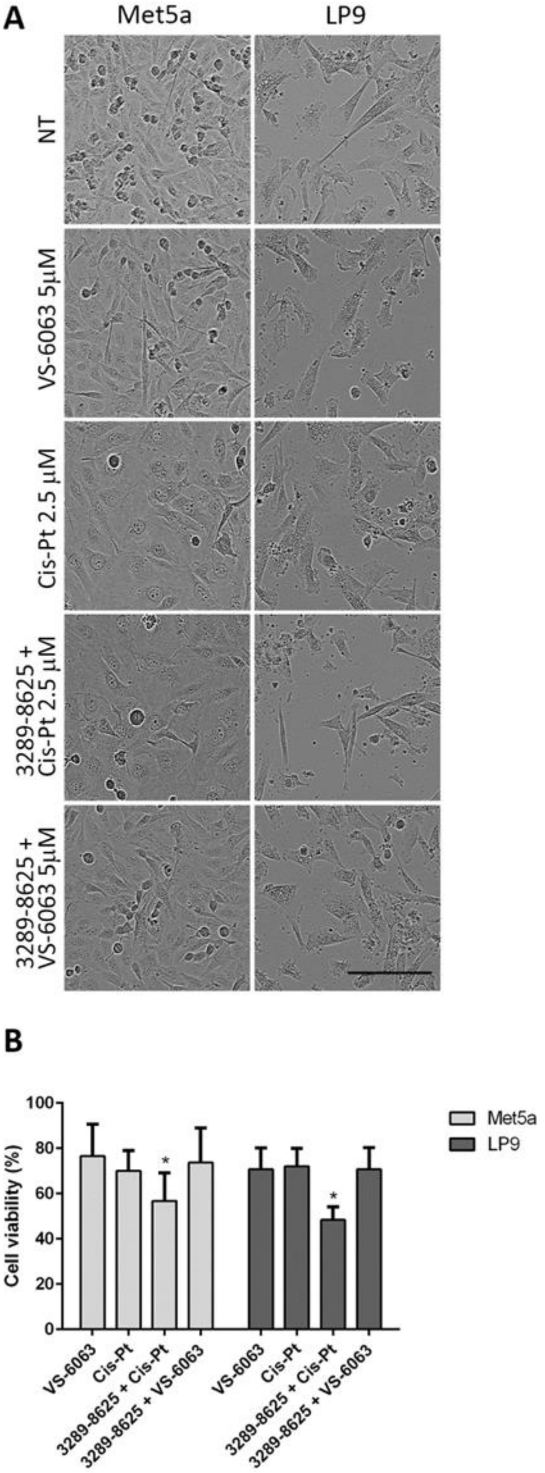

Figure S5. Effect on cell proliferation and survival of treatments with the FAK inhibitor VS-6063, Cis-Pt, the combination of the Wnt inhibitor 3289-8625 + Cis-Pt or 3289-8625 + VS-6063 in the cell lines Met-5a and LP9/TERT-1 (LP9). (**A**) Brightfield images of both immortalized mesothelial cell lines taken 72 h after the start of treatments. Scale bar: 400 µm. (**B**) Cell viability measured with an MTT assay after 72 h of treatments (n = 3 independent experiments; * $p \leq 0.05$). Signals are normalized to non-treated cells.
